# An siRNA Screen of Membrane Trafficking Genes Highlights Pathways Common to HIV-1 and M-PMV Virus Assembly and Release

**DOI:** 10.1371/journal.pone.0106151

**Published:** 2014-09-04

**Authors:** Xiaoyun Wen, Lingmei Ding, Eric Hunter, Paul Spearman

**Affiliations:** 1 Department of Pediatrics, Emory University School of Medicine and Children's Healthcare of Atlanta, Atlanta, Georgia, United States of America; 2 Emory Vaccine Center, Yerkes National Primate Research Center, Emory University, Atlanta, Georgia, United States of America; University of Alabama at Birmingham, United States of America

## Abstract

The assembly and release of retroviruses from the host cells requires a coordinated series of interactions between viral structural proteins and cellular trafficking pathways. Although a number of cellular factors involved in retrovirus assembly have been identified, it is likely that retroviruses utilize additional trafficking factors to expedite their assembly and budding that have not yet been defined. We performed a screen using an siRNA library targeting host membrane trafficking genes in order to identify new host factors that contribute to retrovirus assembly or release. We utilized two retroviruses that follow very distinct assembly pathways, HIV-1 and Mason-Pfizer monkey virus (M-PMV) in order to identify host pathways that are generally applicable in retrovirus assembly versus those that are unique to HIV or M-PMV. Here we report the identification of 24 host proteins identified in the screen and subsequently validated in follow-up experiments as contributors to the assembly or release of both viruses. In addition to identifying a number of previously unsuspected individual trafficking factors, we noted multiple hits among proteins involved in modulation of the actin cytoskeleton, clathrin-mediated transport pathways, and phosphoinositide metabolism. Our study shows that distant genera of retroviruses share a number of common interaction strategies with host cell trafficking machinery, and identifies new cellular factors involved in the late stages of retroviral replication.

## Introduction

Retroviruses assemble capsids and bud from cellular membranes by following one of two distinct morphogenetic pathways [1], [2]. The first pathway is designated the type B/D pathway, in which immature cores assemble in the cytoplasm of cells and subsequently are transported to the plasma membrane for envelopment and budding. The prototypes for the type-B/D pathway are the beta-retroviruses Mason-Pfizer monkey virus (M-PMV) and mouse mammary tumor virus (MMTV). The second pathway is the C-type pathway, in which viral components assemble on the plasma membrane, with simultaneous assembly of the immature capsid shell and acquisition of the lipid envelope. C-type assembly is the most common form of retrovirus assembly, as exemplified by the avian sarcoma/leucosis viruses, murine leukemia viruses, and lentiviruses including human immunodeficiency virus type 1 (HIV-1). The striking difference between the B/D pathway and C-type assembly suggests that there may be unique host elements regulating trafficking of viral components by one path or the other. On the other hand, the process of envelopment and budding of retroviral capsids at the plasma membrane includes common features and is likely to involve host factors that are common to retroviruses following either pathway.

The assembly and release of retroviruses from host cells requires a series of coordinated interactions between the viral structural proteins, the viral genomic RNA, and a wide variety of cellular factors. One of the best-characterized host pathways contributing to retroviral budding is the ESCRT pathway. Retroviral L domains within Gag serve as direct interaction sites for ESCRT components, recruiting a series of components that ultimately leading to the assembly of the ESCRT-III membrane fission complex at the site of viral budding [3]–[6]. Phosphatidylinositol-4,5-bisphosphate interacts with the retroviral MA protein and facilitates HIV-1 Gag interactions with the plasma membrane [7]. The clathrin-related adaptor complexes AP-1, AP-2 and AP-3 have been shown to interact with HIV-1 Gag and participate in its trafficking and release, although the precise mechanisms of action in assembly remain unclear [8]–[11]. Clathrin itself has recently been shown to play a role in retrovirus particle morphogenesis [12]. For M-PMV, the dynein/dynactin motor complex retains Gag in the pericentriolar region of the cells through specific interaction of Tctex-1 with a small peptide motif known as the cytoplasmic targeting-retention signal within MA, facilitating the intracytoplasmic assembly of immature M-PMV capsids [13], . These represent a few examples of the many host factor interactions identified so far that are involved in retrovirus particle assembly.

We hypothesized that many cellular interacting factors involved in retrovirus particle assembly remain to be identified. RNAi analysis is a valuable approach for identifying cellular co-factors necessary for HIV replication. However, the reported siRNA screening studies primarily identified host factors that are required for early stages of the HIV replication cycle and not assembly-related factors [15]–[17]. To gain insight into the mechanism of retrovirus assembly and release, we designed a screen that focuses on the assembly and release and not on early events of the viral lifecycle. We identified 24 host proteins involved in intracellular trafficking that affected both HIV-1 and M-PMV assembly and release. A number of interrelated factors were identified, including those involved in actin cytoskeletal regulation, clathrin-mediated trafficking pathways, and proteins involved in phosphoinositide pathways.

## Results

### siRNA screen

**Figure 1:**
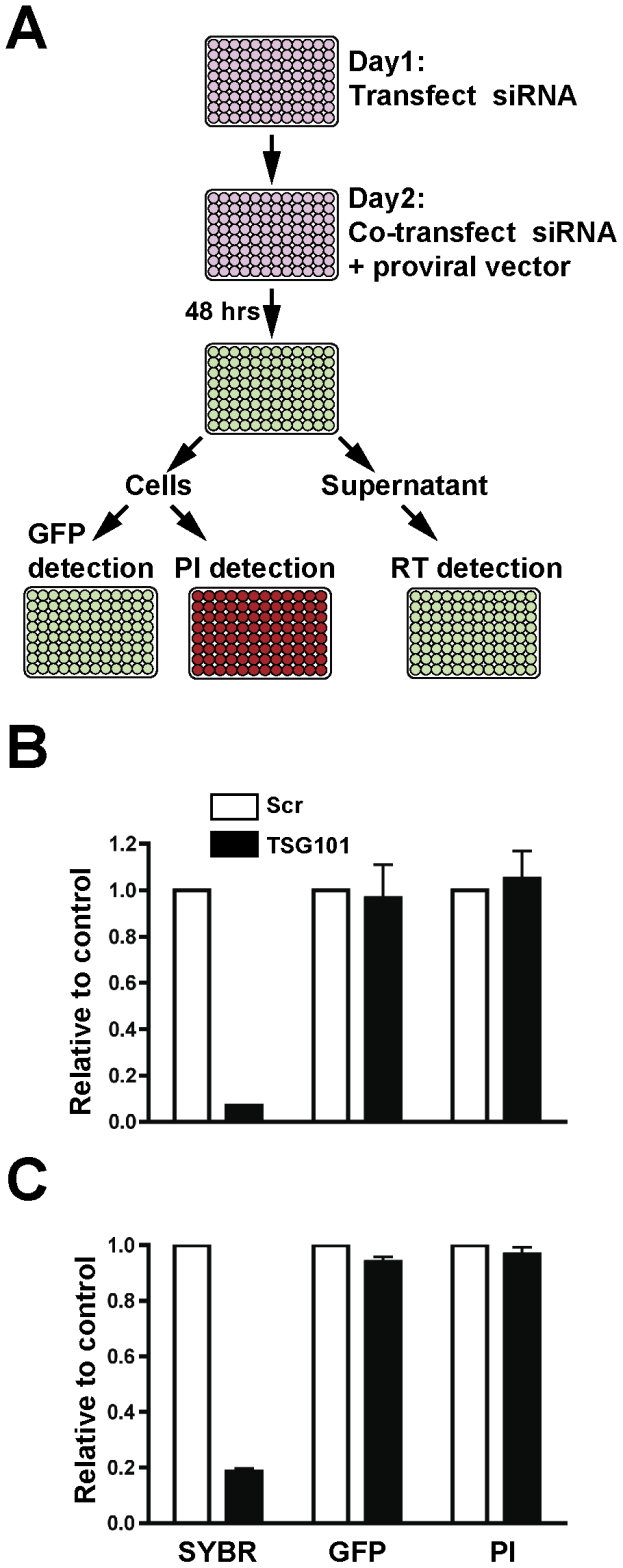
siRNA screen for cellular factors required for HIV-1 and M-PMV assembly and release. (A) Schematic representation of the screen. siRNAs pools were transfected into HeLa or Cos-1 cells on day 1 in a 96-well format. A second transfection with the identical siRNA smartpool together with proviral vector was performed 24 hours after the first transfection. Following an additional 48 hour incubation, particle production in the supernatant was determined by reverse transcription assay. Intracellular GFP expression and cell viability as indicated by PI staining were also measured. (B) siRNA against TSG101 reduces HIV-1 virus output without significantly affecting cell viability and intracellular GFP levels. The scrambled siRNA or TSG siRNA was transfected into HeLa cells with NL4-3-EGFP HIV proviral vector as described above and the effects on HIV particle output, intracellular GFP expression and cell viability were quantified relative to control scrambled siRNA. Error bars indicate the standard deviation of three independent experiments. (C) siRNA against TSG101 reduces M-PMV virus output without significantly affecting cell viability and intracellular GFP levels. The scrambled siRNA or TSG siRNA was transfected into Cos-1 cells with pSARMX-EGFP and pTMO-Env expression vectors as described above and the effects on M-PMV particle output, intracellular GFP expression and cell viability were quantified relative to control scrambled siRNA. Error bars indicate the standard deviation of three independent experiments.

To identify human cellular factors required for HIV-1 and M-PMV assembly and release, we developed an RNAi assay that bypasses early events in the retrovirus lifecycle. Figure 1A shows a schematic representation of the screening strategy employed. HeLa cells were used for monitoring HIV particle assembly and release, while Cos-1 cells were used for M-PMV studies. An siRNA library of 140 membrane trafficking genes each targeted by a pool of four siRNAs (Membrane Trafficking siRNA library, Dharmacon) was employed in the screen. In order to achieve a high knockdown efficiency, the siRNAs were first transfected into cells, followed by a second siRNA transfection together with the proviral vector 24 hours later. HeLa cells were transfected with NL4-3-EGFP provirus, while Cos-1 cells were transfected with an M-PMV-GFP provirus expressing GFP (pSARMX-EGFP) [18]. 48 hours following the second transfection, supernatants were harvested and reverse transcriptase assay assayed using a SYBR green-based readout. To control for transfection efficiency and protein expression, intracellular GFP expression levels were quantified with a fluorescent plate reader. We also monitored cell viability by PI staining to rule out general cytotoxicity. The screen was performed using duplicate wells for each siRNA pool and the entire screen was performed twice. TSG101 siRNA was employed as positive control. TSG101 has been shown to be required for both HIV-1 and M-PMV late budding events [19], [20]. As shown in Fig. 1B and 1C, depletion of TSG101 produced a significant decrease in both HIV-1 and M-PMV viral particle output measured by the SYBR green-based reverse transcriptase assay, as compared with control siRNA-treated cells. The intracellular GFP levels and cell viability indicated by PI staining following TSG101 depletion were comparable to that in control cells.

### Primary siRNA screen

The screen described above was used to detect human cellular factors required for HIV-1 and M-PMV virus assembly and release. The siRNA library targets 140 membrane-trafficking genes (four siRNAs per gene) (Table S1). The siRNAs that inhibited virus particle output by more than 50% were classified as positive “hits”. We discarded results where test siRNAs reduced intracellular GFP expression levels by more than 60% as compared with control siRNA-treated cells, or where PI signal was increased over control transfected cells by more than 60%. These criteria were met by 41 siRNA pools in the HIV-1 screen and 52 pools in the M-PMV screen (Table S2). There were 24 siRNA targets identified by these criteria that were common for both HIV-1 and M-PMV. We next sought ways of analyzing the interrelationships of the factors that were identified, especially those found to be common to both HIV-1 and M-PMV assembly or release, and to perform further validation of selected hits.

### Analysis of interaction networks among identified hits

**Figure 2:**
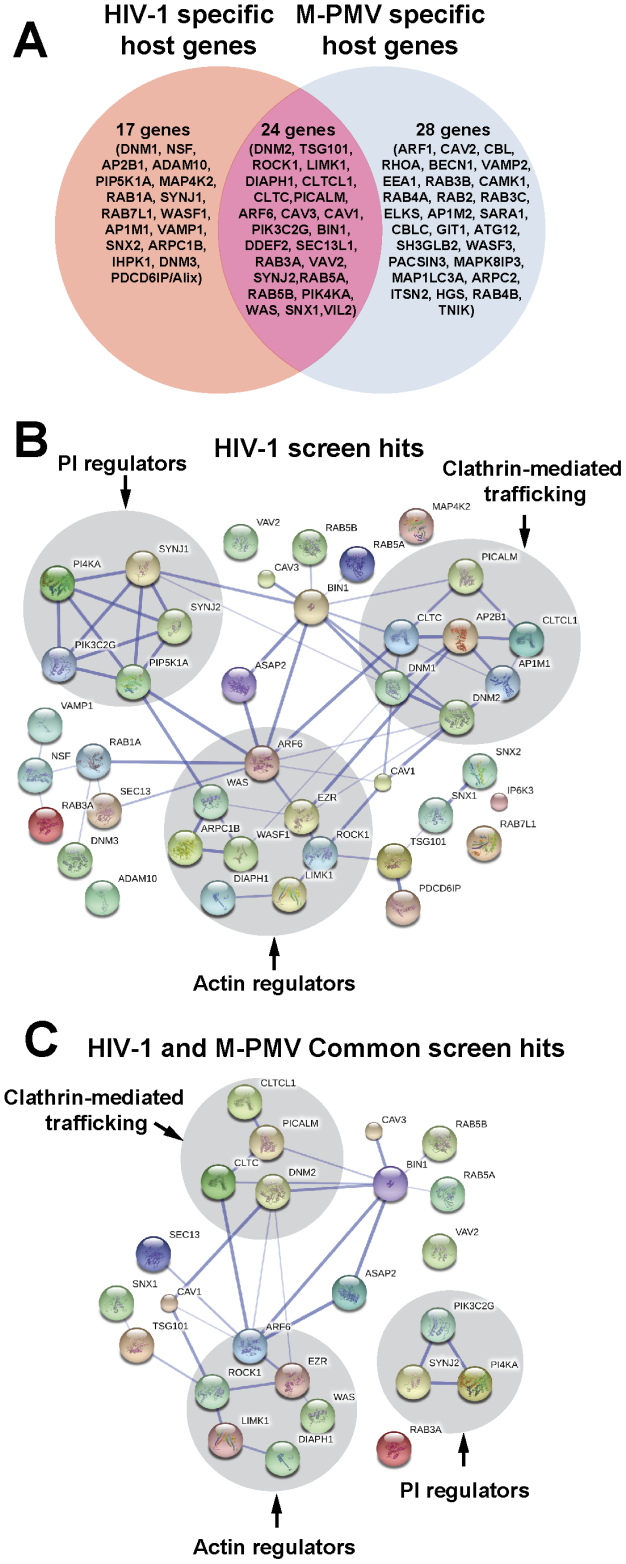
Bioinformatics analyses of siRNA screen hits. (A) Venn diagram of siRNA hits identified in this work. We identified 41 host proteins in HIV screen and 52 host proteins in M-PMV screen. There were 24 host proteins that were common for both HIV-1 and M-PMV. (B) Protein interaction network of HIV1 screen hits (Stronger associations are represented by thicker lines). A map of the interactions of our HIV screen hits was built by using STRING 9.05 with a required medium confidence score (0.400). Analysis of the network revealed clusters of interrelated factors involved in three distinct pathways (highlighted in grey circle): cellular actin cytoskeletal regulation pathways, clathrin-mediated trafficking factors and regulators of phosphoinositide metabolism. (C) Protein interaction network of the common screen hits for both HIV-1 and M-PMV (Stronger associations are represented by thicker lines). The interactions map of 24 host proteins that were common for both HIV-1 and M-PMV was built by using STRING 9.05 with a required medium confidence score (0.400). Factors involved in clathrin-mediated trafficking, regulation of the actin cytoskeleton, and phosphoinositide metabolism (highlighted in grey circle) were identified to affect both HIV-1 and M-PMV assembly or release.

Of the 41 HIV-1 screen hits, eight had been previously directly implicated in HIV assembly, budding, or release: TSG101 [20], , PDCD6IP/ALIX [4], PI4KA [7], AP1M1 [10], AP2B1 [8], RAB7L1 [22], and clathrin heavy chain components (CLTC and CLTCL1) [12]. The factors that were positive only in the HIV-1 screen, the M-PMV screen, or were found to be common to both screens are depicted in Fig. 2A. It was clear upon initial analysis that in addition to those eight factors already identified as playing a role in HIV-1 assembly or release, many additional factors identified in the screen belonged to related pathways (such as multiple factors associated with clathrin-mediated trafficking). However, to provide a more rational analysis of the interrelated factors identified we used the Search Tool for the Retrieval of Interacting Genes (STRING) database and software [23], [24]. STRING is a database that attempts to build interactive networks based upon known protein-protein interactions as well as predicted interactions based upon predicted interactions such as those occurring in sequential steps along a common metabolic pathway. A map of the interactions of our HIV screen hits was built by using STRING 9.05, using a medium confidence analysis according to the published tool (tool available at http://string-db.org). Analysis of the network of HIV screen hits via STRING revealed clusters of interrelated factors involved in three distinct pathways: cellular actin cytoskeletal regulation pathways (WAS, WASF1, ARF6, ARPC1B, ROCK1, LIMK1, DIAPH1, EZR); clathrin-mediated trafficking factors (PICALM, CLTC, AP2B1, CLTCL1, DNM1, DNM2, AP1M1); and regulators of phosphoinositide metabolism (PI4KA, SYNJ1, SYNJ2, PIP5K1A, PIK3C2G). In addition to these three clusters, a number of other relationships between identified trafficking factors and some apparently isolated “hits” were evident as shown in Fig. 2B. In this figure, the width of the connecting lines represents the strength of the association as determined by the STRING program, while the length of the connections and arrangement of the factors for presentation is arbitrary and user-defined.

We next examined the genes identified in both the HIV-1 and M-PMV screens to evaluate potential common pathways involved in the late phases of retroviral replication. Notably, the same three major groupings seemed to hold in this analysis. Factors involved in clathrin-mediated trafficking, regulation of the actin cytoskeleton, and phosphoinositide metabolism were identified as important for HIV-1 and M-PMV assembly or release (Fig. 2C).

### Validation by short hairpin RNA

**Figure 3:**
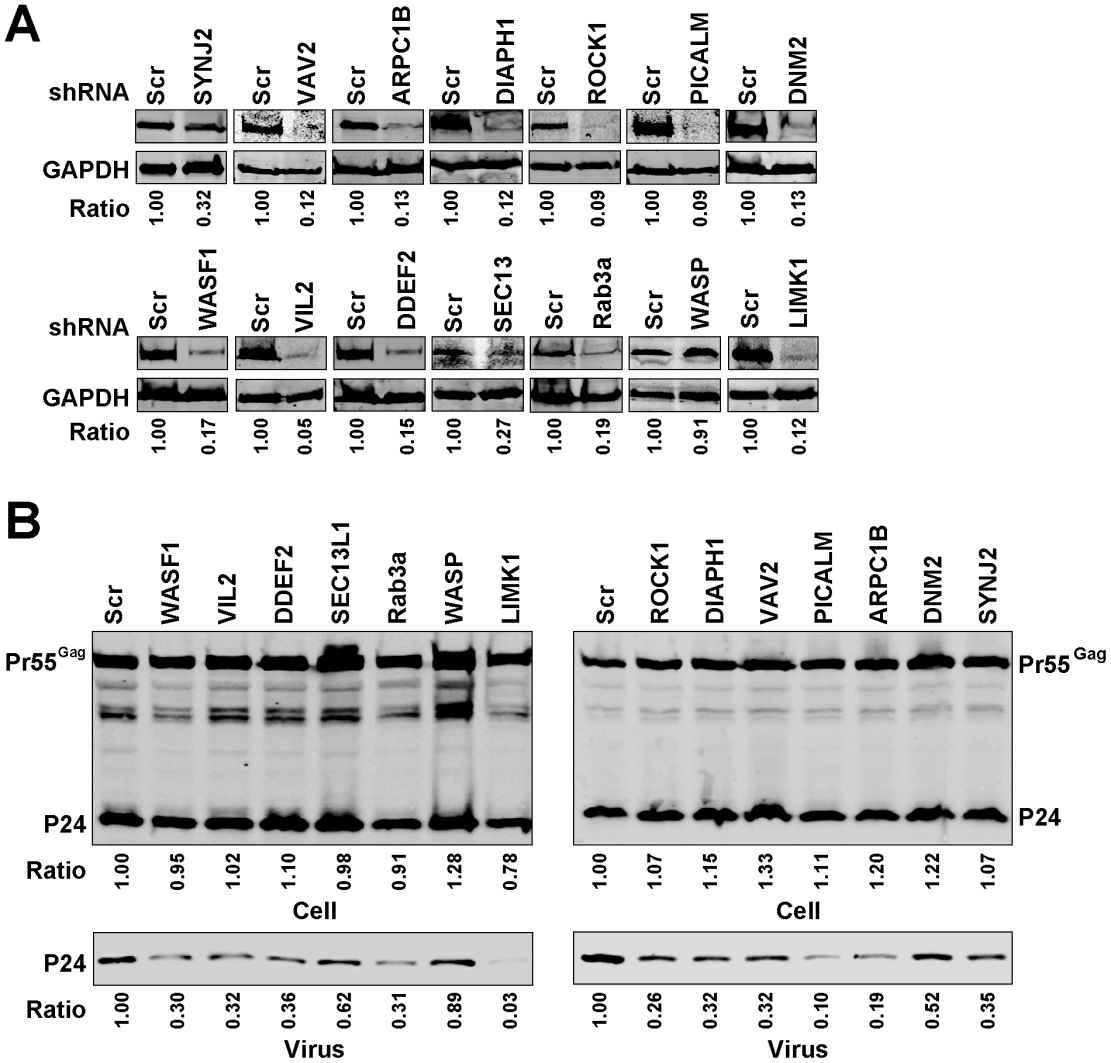
Effect of silencing the selected genes on HIV1 particle output. (A) Quantitative western blot analysis of knockdown efficiency of the individual genes in HeLa cells. HeLa cells stably expressing control shRNA or specific shRNAs against each individual gene were generated and the cellular levels of the individual protein were examined. The ratio of the individual protein levels as quantified on the LiCor Odyssey compared to control cell is shown below each blot. (B) The effect of depleting the individual genes on HIV-1 particle output. HeLa cells stably expressing control shRNA or the indicated shRNAs were transfected with NL4-3 HIV proviral vector. At 48 hours after transfection, the virions in the supernatant and cell lysates were harvested and subjected to immunoblotting with p24 antibody. The ratio of supernatant p24 and the ratio of the total amount of cellular p24 and p55 as quantified on the LiCor Odyssey compared to control cells are shown below each blot.

siRNA screens may identify false hits due to off-target effects of the siRNA or through introduction of cellular toxicities that can mimic a specific effect in the readout assay. In our screen, despite some validation through the identification of eight known factors involved in HIV assembly or release, we were concerned with the relatively large number of genes identified as important for M-PMV and HIV particle production. We chose fourteen of the identified genes (related either to actin regulation, clathrin-mediated trafficking, or other aspects of vesicular trafficking) to validate in further experiments using short hairpin RNA (shRNAs). We generated HeLa cells stably expressing control shRNA or specific shRNAs against each individual gene. Knockdown efficiency was assessed by Western blot analysis, and varied from 90–95% as shown in Figure 3A, with one exception of *WAS* for which we were unable to achieve a stably-depleted cell population (shown with WASP label on Fig. 3A and 3B). Each depleted cell population was then transfected with NL4-3, and particle output assessed by Western blotting (Fig. 3B). Consistent with our HIV screen results, we observed a reduction in HIV-1 particle output following the depletion of each of the 13 individually depleted genes, with WAS/WASP cells serving as an additional non-depleted control. Depletions of the gene products SYNJ2, VAV2, ARPC1B, DIAPH1, ROCK1, PICALM, WASF1, VIL2, DDEF1, and Rab3a caused similar levels of reduction (10–36% of control) of p24 output. The depletion of Lim kinase (LIMK1) had the largest impact on HIV-1 particle output, whereas depletion of dynamin 2 (DNM2) and SEC13-like protein 1 (SEC13L1) only caused modest reductions in HIV-1 particle output. Cell lysates and supernatants in the same experiment were also assayed for viral p24 content by p24 ELISA, and results were expressed as percentage Gag release (supernatant p24/(supernatant + cellular p55/p24)) normalized to control shRNA-expressing cells (Fig. 5A). Consistent with the previous findings, HIV particle release from LIMK1-depleted HeLa cells was decreased by about 5 fold as compared with control cells, whereas depletion of DNM2 only reduced HIV1 release by about 30%. Depletion of the other ten genes caused a reduction varying from 45% to 65% in HIV particle release by the particle release ratio (Fig. 5A). We note that while the absolute magnitude of the effect observed varied somewhat when measured by Western blot vs. ELISA, the relative inhibitory effects of depletion (such as the strong effect of LIMK1 depletion) were consistently observed for either readout.

**Figure 4:**
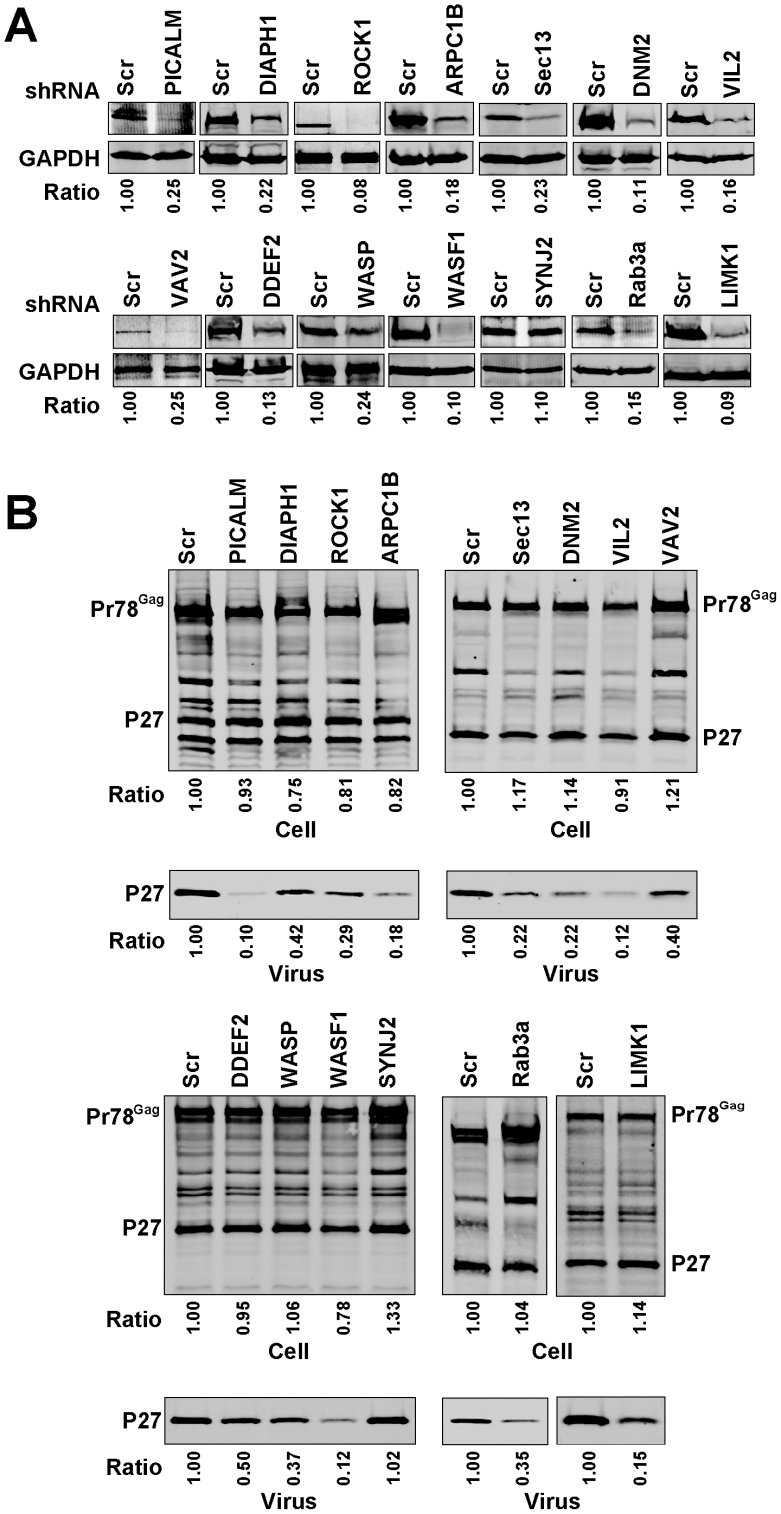
Effect of silencing the selected genes on M-PMV particle output. (A) Quantitative western blots showing knockdown efficiency of the individual genes in Cos-1 cells. Cos-1 cells stably expressing control shRNA or specific shRNAs against each individual gene were generated and the cellular levels of the individual protein were examined. The ratio of the individual protein levels as quantified on the LiCor Odyssey compared to control cell is shown below each blot. (B) The effect of depleting the individual genes on M-PMV particle output. Cos-1 cells stably expressing control shRNA or the indicated shRNAs were transfected with pSARMX M-PMV proviral vector. At 48 hours after transfection, the virions in the supernatant and cell lysates were harvested and subjected to immunoblotting with p27 antibody. The ratio of supernatant p27 and the ratio of the total amount of cellular p27 and Pr78^Gag^ as quantified on the LiCor Odyssey compared to control cells are shown below each blot.

**Figure 5:**
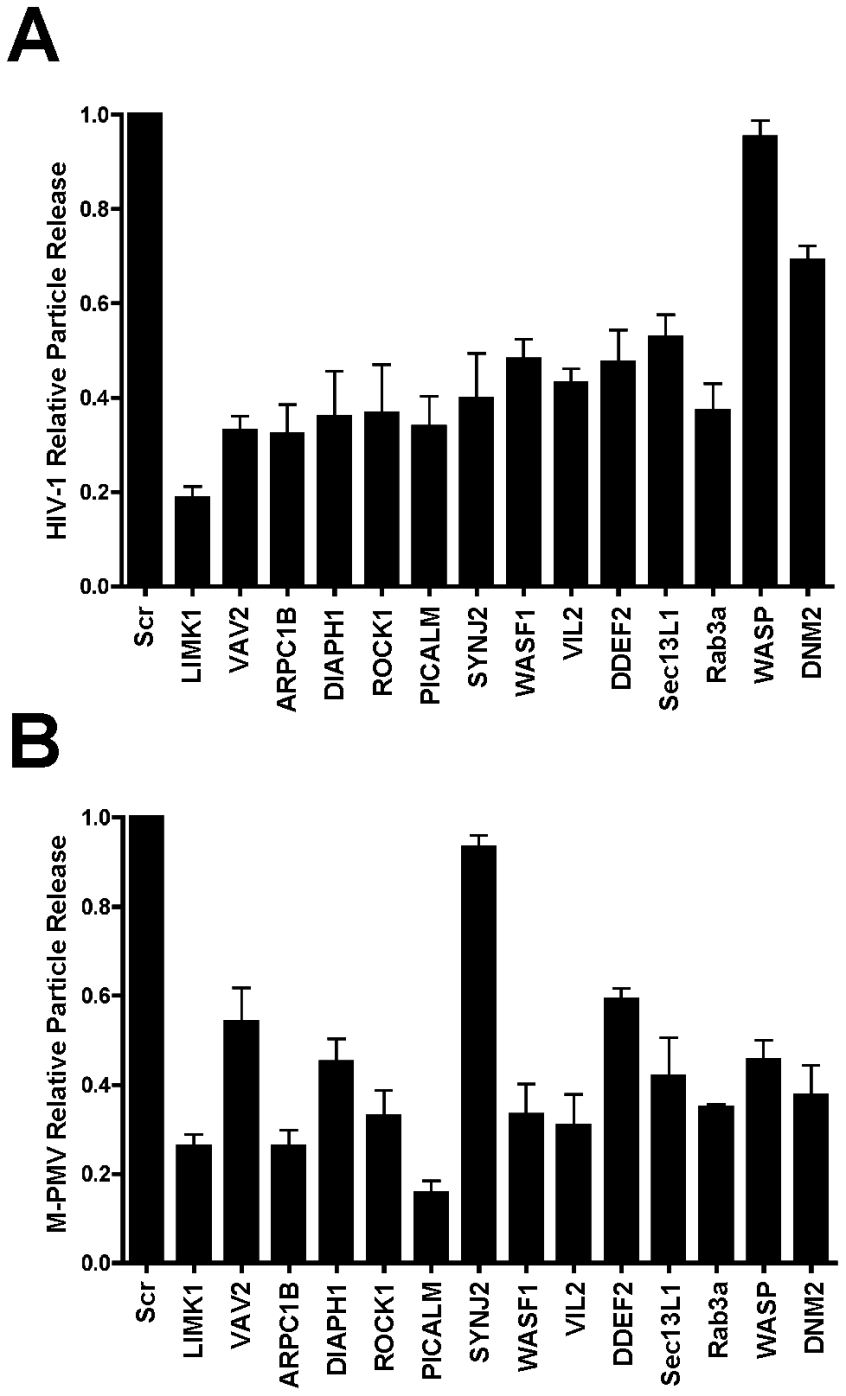
Quantitative analysis of the effect of silencing the selected genes on HIV-1 and M-PMV particle release. (A) The effect of depleting the individual genes on HIV-1 particle release. HIV1 particle release in the control shRNA or specific shRNA expressing HeLa cells was assessed using a p24 antigen ELISA. Results were expressed as percentage Gag release (supernatant p24/(supernatant + cellular p55/p24)) normalized to control shRNA-expressing cells. Error bars indicate the standard deviation of three independent experiments. (B) Quantitative analysis of the effect of depleting the individual genes on M-PMV particle release. M-PMV particle release in the control shRNA or specific shRNA expressing Cos-1 cells was assessed by immunoblotting with p27 antibody. The supernatant p27 and cellular p27 and Pr78^Gag^ were quantified on the LiCor Odyssey. Results were expressed as percentage Gag release (supernatant p27/(supernatant + cellular Pr78^Gag^/p27)) normalized to control shRNA-expressing cells. Error bars indicate the standard deviation of three independent experiments. A representative of 3 independent experiments is shown in Fig 4B.

We next examined the effect of independent depletion of the same fourteen genes in COS-1 cells on M-PMV particle output. Knockdown efficiency in COS-1 cells assessed by Western blot analysis was shown in Figure 4A and varied from 85–95%, with one exception of *SYNJ2*, which was not significantly depleted. At 48 hours after the transfection of M-PMV pSARMX provirus vector, P27 antigen levels in the cell lysates and supernatants were examined by Western blot analysis (Fig. 4B). We observed that M-PMV particle output was significantly reduced by depletion of PICALM, ROCK1, ARPC1B, Sec13L1, DNM2, VIL2, WASF1, Rab3a or LIMK1, and to a lesser extent, by depletion of DIAPH1, VAV2, DDEF1, or WASP. The ratio of supernatant p27 as quantified on the LiCor Odyssey compared to control cell supernatant is shown below each blot. To present these data graphically, we then derived a relative particle release value of supernatant p27 output adjusted for total Pr78^Gag^ levels as compared to control cells in triplicate experiments (Fig. 5B). These results confirmed an effect on particle output of M-PMV by each of the thirteen factors for which we achieved a significant knockdown, and the lack of SYNJ2 knockdown served as an internal control with near-wildtype levels of output. We conclude that based upon these shRNA validation studies, our screen correctly identified a series of factors that are involved in HIV-1 and M-PMV assembly or release.

**Figure 6:**
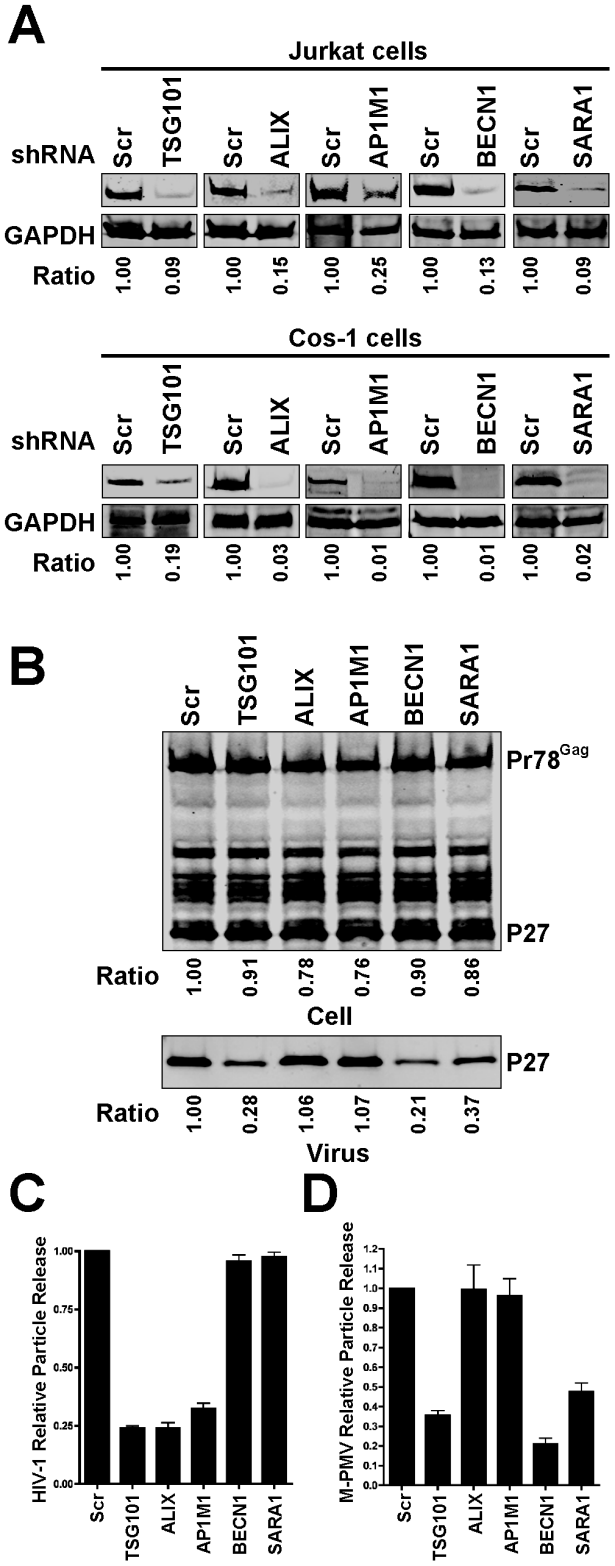
Effect of silencing of TSG101, ALIX1, AP1M1, BECN1 and SARA1 on HIV-1 and M-PMV particle output. (A) Quantitative western blots showing knockdown efficiency of the individual genes in Jurkat cells and Cos-1 cells. Jurkat cells and Cos-1 cells stably expressing control shRNA or specific shRNAs against individual genes were generated, and the cellular levels of the individual proteins examined by immunoblotting. The ratio of the individual protein levels as quantified on the LiCor Odyssey compared to control cell is shown below each blot. (B) The effect of depleting the individual genes on M-PMV particle output. Cos-1 cells stably expressing control shRNA or the indicated shRNAs were transfected with pSARMX M-PMV proviral vector. At 48 hours after transfection, the virions in the supernatant and cell lysates were harvested and subjected to immunoblotting with p27 antibody. The ratio of supernatant p27 and the ratio of the total amount of cellular p27 and Pr78^Gag^ as quantified on the LiCor Odyssey compared to control cells are shown below each blot. (C) The effect of depleting the individual genes on HIV-1 particle release in Jurkat cells. Control shRNA or specific shRNA expressing Jurkat cells were infected with vesicular stomatitis virus G glycoprotein (VSV-G)-pseudotyped HIV-NL4-3 virus (at 1 TCID_50_) overnight at 37°C, washed and then seeded in 12-well plates. HIV-1 particle release was assessed using a p24 antigen ELISA 2 days post-infection. Results were expressed as percentage Gag release (supernatant p24/(supernatant + cellular p55/p24)) normalized to control shRNA-expressing cells. Error bars indicate the standard deviation of three independent experiments. (D) Quantitative analysis of the effect of depleting the individual genes on M-PMV particle release. M-PMV particle release in the control shRNA or specific shRNA expressing Cos-1 cells was assessed by immunoblotting with p27 antibody. The supernatant p27 and cellular p27 and Pr78^Gag^ were quantified on the LiCor Odyssey. Results were expressed as percentage Gag release (supernatant p27/(supernatant + cellular Pr78^Gag^/p27)) normalized to control shRNA-expressing cells. Error bars indicate the standard deviation of three independent experiments.

In order to further validate the assembly screen performed here, we performed follow-up depletion experiments of a subset of the identified host factors that uniquely altered assembly or release of either HIV-1 or M-PMV and not both. Since TSG101 has been reported to be required for both HIV-1 and M-PMV particle output, we included this as a positive control in this analysis [19], [20]. We generated both Jurkat cells and Cos-1 cells stably expressing control shRNA or specific shRNAs against each individual gene. Knockdown efficiency was assessed by Western blot analysis, and varied from 75–91% in Jurkat cells and from 81–99% in Cos-1 cells as shown in Figure 6A. Cos-1 cells were transfected with pSARMX and M-PMV particle output monitored by Western blot. We observed that M-PMV particle output was significantly reduced by depletion of TSG101, BECN1, or SARA1, but not by depletion of ALIX or AP1M1 (Fig. 6B). Particle output data is presented graphically in Fig. 6D (supernatant p27 output adjusted for total Pr78^Gag^ levels in triplicate experiments). Next, Jurkat cells stably expressing control shRNA or specific shRNAs against individual genes were infected with VSV-G-pseudotyped HIV-NL4-3 virus, and HIV-1 particle release assessed using a p24 antigen ELISA 2 days post-infection. Consistent with our previous siRNA screen results, depletion of TSG101, ALIX or AP1M1 significantly reduced HIV-1 particle release (Fig. 6C), while depletion of BECN1 or SARA1 had little effect on HIV-1 particle release (Fig. 6C). These results support the specific findings of the screening strategy, and indicate that there are shared host factors involved in HIV-1 and M-PMV assembly and release, as well as host factors that are unique to assembly or release of one virus and not the other.

## Discussion

Retroviruses depend upon interactions with a variety of cellular factors to achieve productive replication. Identifying those cellular factors that retroviruses usurp for their own needs can provide important new insights into basic virology, and may illuminate new targets for antiretroviral therapy. The use of small-interfering RNA (siRNA) screens has greatly extended our knowledge of the cellular factors hijacked by viruses for productive replication. Here we utilized a directed siRNA screen using a library greatly enriched in factors involved in intracellular trafficking pathways. To identify host factors associated with HIV and M-PMV assembly and release, we performed an siRNA screen that bypassed early events in the virus lifecycle and focused on particle production and release. We identified 24 host proteins that upon depletion diminish both HIV-1 and M-PMV assembly or release. Common to the production of both retroviruses were gene products involved in actin cytoskeletal regulation, clathrin-mediated trafficking pathways, and phosphatidylinositol regulation. The screen also identified individual trafficking factors that have not been connected directly with retroviral particle production and are worthy of further investigation. Eight gene products previously identified as playing important roles in late events of the HIV-1 lifecycle, including TSG101 and ALIX, were identified and help establish the validity of the screening methodology. To further validate the findings we performed directed shRNA depletion experiments on a subset of the identified hits. Although we did not validate all hits from the screen, the directed evaluation of fourteen identified hits was supportive of the findings in the siRNA screen.

A number of genome-wide screens for host factors involved in HIV replication have been performed in recent years [15]–[17], [25]–[27]. Factors relevant to assembly, budding, and release have been relatively under-represented in the hits from these screens, with some notable exceptions. We noted several factors identified in our screen that have been found via previous screening methods. ADAM10 was identified in two previous screens [16], [26] as a host factor involved in HIV-1 replication. However, its role has been described as a facilitator of nuclear trafficking [28], which would not fit with the assembly/release screening methodology employed here. Its role as a metalloprotease acting at the plasma membrane could potentially enhance particle release and deserves further evaluation. Phosphatidylinositol 4-kinase (PI4KA) was previously identified in the Zhou et al. genome-wide screen [15], and fits well with established models of HIV-1 assembly as this kinase catalyzes the biosynthesis of phosphatidylinositol 4,5-bisphosphate (PI(4,5)P_2_). PI(4,5)P_2_ is found on the inner leaflet of the plasma membrane and interacts with HIV-1 MA during the assembly process [7], . The role of PI(4,5)P_2_ in M-PMV assembly events is less certain, as it appears unable to trigger the myristyl switch for M-PMV MA [30]. One of the most potent host factors implicated in our current study was LIMK1, which was validated in shRNA knockdown experiments to affect both HIV-1 and M-PMV particle production. LIMK1 was not identified in the prior genome-wide screens. However, Konig et al. identified LIMK2, a closely-related kinase that is activated by ROCK1 and phosphorylates cofilin, in their screen [27].

It has long been proposed that the actin cytoskeleton is involved in retrovirus production, primarily through observational data. Actin and actin-related proteins are highly represented in HIV-1 virions [31], [32], although the specificity and significance of this finding has been questioned [33]. Cryo-electron tomography analysis of HIV-1 assembly sites revealed that half of the HIV budding sites in their study were present on actin-filled filopodia where actin filaments were directed arranged towards the budding sites [34]. Actin-disrupting agents (e.g. cytochalasin D and latrunculin B) have been applied to retrovirus-infected cells and generally led to a decrease in virus release of only about 50%, thus failing to conclusively demonstrate a role for actin polymerization in this type of experiment [35]. We identified several cellular actin cytoskeleton-associated host factors that may be important for HIV particle output. *WAS* and *WASF1* belong to the Wiskott-Aldrich syndrome protein (WASP) family that plays a critical role in regulating the actin cytoskeleton required for membrane ruffling [36]. WAS has been shown to associate with the actin nucleation core ARP2/3 complex [37]. ARPC1B was identified in our HIV-1 screen, is one of seven subunits of ARP2/3 complex. Data from the present screen are thus supportive of a role for actin nucleation in the late events of the retrovirus lifecycle. A second pathway of interest in this regard is the Rho-ROCK1-LIMK1-Cofilin pathway. Once activated by upstream kinases including ROCK1, LIMK1 promotes actin polymerization by phosphorylating and inactivating the actin depolymerizing factor Cofilin. The identification of ROCK1 and LIMK1 as candidate factors regulating HIV-1 and M-PMV assembly or release is intriguing and led us to evaluate this pathway further. During the review of this manuscript, we published a more in-depth analysis of the role of LIMK1 in retrovirus particle release [38]. Unexpectedly, disruption of the ROCK1-LIMK1 pathway resulted in a very late defect in particle release following budding that was common to both HIV-1 and M-PMV [38].

The role of inositol phosphates in retrovirus assembly is incompletely understood. Ono *et al.* has shown that PI(4,5)P_2_ regulates HIV-1 Gag targeting to the plasma membrane [7]. An *in vitro* study by Campbell *et al.* showed that the interaction between HIV-1 Gag protein molecules are altered by binding of inositol derivatives and this binding is essential for normal HIV-1 particle assembly [39]. We identified three PI kinases, PIK3C2G, PI4KA, and PIP5K1A, as candidate factors involved in HIV-1 assembly, and two were picked up in both screens (PIK3C2G and PI4KA). These three PI kinases may be involved in regulating inositol phosphates in specific membrane microdomains or in regulating trafficking of Gag, and also warrant further investigation.

Our study has inherent limitations. We screened a relatively small, focused library that is already enriched in factors likely to be involved in some aspect of enveloped virus assembly. The number of positive hits in such a limited library was concerning, but validation through shRNA depletion in repeated experiments targeting a subset of the identified host factors was reassuring. Overall we believe the study has two major aspects of significance. First, it identifies a number of candidate host trafficking factors that can be pursued in follow-up studies and which may reveal new mechanistic insights into retrovirus assembly or release. Second, and perhaps more importantly, the clustering of identified factors into those associated with clathrin-mediated trafficking, regulation of actin dynamics, and regulation of phosphoinositides is striking. We suggest that detailed studies of the role each of these three general pathways play in retrovirus assembly are likely to be quite revealing.

## Materials and Methods

### Cells

HeLa (ATCC CCL-2) and Cos-1 cells (ATCC CRL-1650) were obtained from the American Type Culture Collection (ATCC), and were maintained in Dulbecco's modified Eagle's medium (DMEM) containing 10% FBS and antibiotics at 37°C with 5% CO_2_. Jurkat T cells (ATCC TIB-152) were obtained from ATCC and cultured in RPMI medium 1640 supplemented with 10% FBS, 2 mM Glutamine, and antibiotics.

### RNAi Screen

To identify host factors required for HIV-1 and M-PMV assembly and release, a RNAi-based screen was performed using a commercially-available library targeting 140 cellular membrane-trafficking genes (Dharmacon-Thermo Fisher Scientific; Table S1). HeLa or Cos-1 cells were plated in 96-well tissue culture plates and all transfections were performed using Lipofectamine 2000 (Invitrogen, Carlsbad, CA, USA) according to manufacturer's instructions. In order to achieve a high knockdown efficiency, siRNAs were transfected into HeLa or Cos-1 cells at a 60 nM final concentration on day 1. A second transfection with the identical siRNA smartpool together with 0.4 ug NL4-3-EGFP (HeLa) or 0.2 ug pSARMX-EGFP and 0.2 ug pTMO-Env (Cos-1) [18] expression vectors was performed 24 hours after the first transfection. Following an additional 48 hour incubation, the plates were spun down at 4,500 RPM for 5 minutes at 4°C and then supernatants were harvested. Particle production was measured using EnzChek reverse transcriptase assay kit (Molecular Probes, Eugene, OR, USA) according to manufacturer's protocol, but in order to increase the signal, we added 20 ul SYBR green PCR master mix (Applied Biosystems, California, USA) to each well instead of the PicoGreen reagent included in the kit and had the plates read on real-time PCR machine (7500 Fast Real-Time PCR system, Applied Biosystems). The intracellular GFP reporter gene expression was measured using a Perkin-Elmer Envision 2013 multilabel reader (Ex filter 485 nm, Em filter 535 nm). Cell viability was determined by PI staining and assessed with the Envision plate reader (Ex filter YFP 535 nm, Em filter Europium 615 nm).

### shRNA constructs

ShRNA constructs were purchased from Sigma-Aldrich (St. Louis, MO, USA). The specific constructs are listed in Table S3.

Control shRNA or targeted shRNA-expressing pseudoviruses were generated by cotransfecting 293T cells with the shRNA vectors (4–5 shRNAs per gene) and pHCMV-G [40]. The shRNA was delivered into target cells following the manufacturer's recommendations, using the pLKO.1-puro backbone vector. For transduction, HeLa, Jurkat and Cos-1 cells were incubated with the shRNA lentiviral stock overnight. 24 hours post-transduction, the medium was aspirated and replaced with fresh medium containing puromycin at 2 ug/ml for HeLa or 4 ug/ml for Jurkat and Cos-1. After 1 week of puromycin selection, the cells were ready for experiments.

### Virus stocks and infections

Vesicular stomatitis virus G glycoprotein (VSV-G)-pseudotyped HIV-NL4-3 was generated by transfecting 293T cells. On day 2 post transfection, viruses were harvested from the supernatant, filter-sterilized and assayed with TZM-β1 indicator cells for infectivity assessment. Jurkat cells were infected with VSV-G-pseudotyped HIV-NL4-3 virus (at 1 50% tissue culture infectious dose (TCID_50_)) for overnight at 37°C, washed and then seeded in 12-well plates.

### Western Blot Analysis

Puromycin-selected HeLa or COS-1 cells were seeded in 6-well plates and transfected with 0.3 ug NL4-3 (HeLa) or 1 ug pSARMX proviral vector using Lipofectamine 2000 (Invitrogen, Carlsbad, CA, USA) according to manufacturer's instructions. On day 2 post-transfection, cell pellets and culture supernatants were harvested. Culture supernatants were clarified by low speed centrifugation and filtered through a 0.2 µm filter, then layered onto a 20% sucrose cushion in PBS and centrifuged at 20,000 g for 2 hours at 4°C using Sorvall Discovery M-150 SE micro-ultracentrifuge (Thermo Electron Corporation, Waltham, MA, USA). Virion pellets and corresponding cell pellets were dissolved in Laemmli sample buffer (Bio-Rad, Hercules, CA, USA). Virion and cell lysates were separated on 12% polyacrylamide gels and subjected to Western blotting. HIV-1 Gag detection was performed with mouse anti-p24 monoclonal CA-183 antibody (provide by Bruce Chesebro and Kathy Wehrly through the NIH AIDS Research and Reference Reagent Program). Rabbit anti-p27 polyclonal antibody [41]was employed for detection of M-PMV Gag. IRDye goat anti-mouse and IRDye goat anti-rabbit secondary antibodies used for Western blots were obtained from Li-cor Biosciences (Lincoln, NE, USA). Blots were developed and analyzed using the Li-Cor Odyssey infrared detection system.

### Protein Interrelationship Analysis

The protein interaction network was constructed using STRING 9.05 (Search Tool for the Retrieval of Interacting Genes/Proteins) [23], supplemented with functional information from the literature. To view relationships, we utilized the confidence view with a required medium confidence score (0.400).

## Supporting Information

Table S1 **Table of genes included in Thermo Scientific Dharmacon siGenome siRNA Library-Membrane Trafficking.**
(XLS)

Table S2 **Table S2 provides a list of all the identified 69 genes in both HIV screen and M-PMV screen that qualified the selection criteria.** Column A: gene name. Column B: gene ID. Column C: Genbank accession number. Column D: description of the gene name. Column E and G show percent of RT value versus control scrambled siRNA after normalization by intracellular GFP levels in HIV or M-PMV screen when the indicated genes were silenced with pooled siRNAs. Columns F and H provide the corresponding standard deviation (SD) of four experiments. Column I, J, K are GO (gene ontology, http://www.geneontology.org/GO.doc.shtml) classification of molecular function, cellular component and PANTHER protein class of each gene, using the gene ontology PANTHER classification system. ‘Undetectable’ indicates the amount of reverse transcriptase in the sample is below the method detection limit. ‘N/A’ indicates not positive hits in the screen.
(XLS)

Table S3 **Table S3 provides the shRNA constructs utilized in this manuscript as indicated by TRCN clone number.**
(DOCX)
