# Supplementary material for: An siRNA Screen of Membrane Trafficking Genes Highlights Pathways Common to HIV-1 and M-PMV Virus Assembly and Release
Source: PLoS One. 2014 Sep 4;9(9):e106151. doi: 10.1371/journal.pone.0106151 (PMC4154853; doi:10.1371/journal.pone.0106151)
Supplement: Table S3 — provides the shRNA constructs utilized in this manuscript as indicated by TRCN clone number. (DOCX) [file pone.0106151.s003.docx]

| **Table S3. shRNA constructs employed, by TRCN clone number** | |
| --- | --- |
| **Genes** | **Specific shRNA constructs** |
| SYNJ2 | TRCN0000050373, TRCN0000050374, TRCN0000050375, TRCN0000050376, TRCN0000050377 |
| VAV2 | TRCN0000048223, TRCN0000048224, TRCN0000048225, TRCN0000048226, TRCN0000048227 |
| ARPC1B | TRCN0000036494, TRCN0000036495, TRCN0000036496, TRCN0000036497, TRCN0000036498 |
| DIAPH1 | TRCN0000118677, TRCN0000118678, TRCN0000118679, TRCN0000118680, TRCN0000118681 |
| ROCK1 | TRCN0000002159, TRCN0000002160, TRCN0000002161, TRCN0000002162, TRCN0000002163 |
| PICALM | TRCN0000119072, TRCN0000119073, TRCN0000119074, TRCN0000119075 |
| DNM2 | TRCN0000006648, TRCN0000006650, TRCN0000006651, TRCN0000006652 |
| VIL2 | TRCN0000062458, TRCN0000062459, TRCN0000062460, TRCN0000062461, TRCN0000062462 |
| DDEF2 | TRCN0000029749, TRCN0000029750, TRCN0000029752, TRCN0000029753 |
| Sec13L1 | TRCN0000064984, TRCN0000064985, TRCN0000064986, TRCN0000064987 |
| Rab3a | TRCN0000047953, TRCN0000047954, TRCN0000047955, TRCN0000047956, TRCN0000047957 |
| WASP | TRCN0000029819, TRCN0000029820, TRCN0000029821, TRCN0000029822, TRCN0000029823 |
| LIMK1 | TRCN0000000825, TRCN0000000826, TRCN0000010553, TRCN0000010554, TRCN0000010555 |
| WASF1 | TRCN0000122994, TRCN0000122995, TRCN0000122996, TRCN0000122997, TRCN0000122998 |
